# Method of Evaluating the Positioning System Capability for Complying with the Minimum Accuracy Requirements for the International Hydrographic Organization Orders

**DOI:** 10.3390/s19183860

**Published:** 2019-09-06

**Authors:** Mariusz Specht

**Affiliations:** Department of Transport and Logistics, Gdynia Maritime University, Morska 81-87, 81-225 Gdynia, Poland; m.specht@wn.umg.edu.pl

**Keywords:** reliability model, Navigation Satellite System (NSS), positioning accuracy, positioning availability, hydrography

## Abstract

According to the IHO (International Hydrographic Organization) S-44 standard, hydrographic surveys can be carried out in four categories, the so-called orders—special, 1a, 1b, and 2—for which minimum accuracy requirements for the applied positioning system have been set out. These amount to, respectively: 2 m, 5 m, 5 m, and 20 m at a confidence level of 0.95. It is widely assumed that GNSS (Global Navigation Satellite System) network solutions with an accuracy of 2–5 cm (*p* = 0.95) and maritime DGPS (Differential Global Positioning System) systems with an error of 1–2 m (*p* = 0.95) are currently the two main positioning methods in hydrography. Other positioning systems whose positioning accuracy increases from year to year (and which may serve as alternative solutions) have been omitted. The article proposes a method that enables an assessment of any given navigation positioning system in terms of its compliance (or non-compliance) with the minimum accuracy requirements specified for hydrographic surveys. The method concerned clearly assesses whether a particular positioning system meets the accuracy requirements set out for a particular IHO order. The model was verified, taking into account both past and present research results (stationary and dynamic) derived from tests on the following systems: DGPS, EGNOS (European Geostationary Navigation Overlay Service), and multi-GNSS receivers (GPS/GLONASS/BDS/Galileo). The study confirmed that the DGPS system meets the requirements for all IHO orders and proved that the EGNOS system can currently be applied in measurements in the orders 1a, 1b, and 2. On the other hand, multi-GNSS receivers meet the requirements for order 2, while some of them meet the requirements for orders 1a and 1b as well.

## 1. Introduction

To navigate safely or carry out positioning-related surveys (hydrographic or land), it is advisable to be equipped with a positioning system that complies with all operational characteristics assigned to a specific navigation task at the same time. These include: accuracy, operating zone, availability, reliability, continuity, and integrity. These requirements are defined in the radionavigation plans [1,2,3,4], other recommendations or standards related to a specific activity that require positioning, e.g., hydrography [5], railway engineering [6], aviation [7], and land [8] or marine navigation [9]. An analysis of these documents indicated that the number of navigation system applications is increasing each year; however, in terms of the numerical value, the minimum requirements for positioning accuracy and other characteristics assigned to specific applications have remained unchanged over the years.

Contrary to the positioning accuracy requirements that have generally been constant over the years, radionavigation positioning systems (particularly GNSS (Global Navigation Satellite System)) continue to increase their positioning accuracy with the result that they can be used in a particular application for which they were not suitable only a few years ago due to the insufficient positional precision. For example, over the last several years, the GPS (Global Positioning System) has continually enhanced the positioning accuracy and other operational characteristics. In 1993, the accuracy of this system on the horizontal plane was 100 m (*p* = 0.95) [10]. In 2001, after selective availability (SA) was turned off, it increased to 13 m (*p* = 0.95) [11]. However, in 2008 it reached 9 m (*p* = 0.95) [12]. The increase in positioning accuracy has resulted in an increase in the number of applications for the system. For example, in 1993, the system still failed to meet the positioning requirements for car navigation due to the accuracy being too low (100 m, *p* = 0.95), while in 2001 it could be successfully used in this application, which required an accuracy of a few meters. A similar increase in positioning accuracy has been observed for all GPS augmentation systems [13,14,15,16,17].

However, the greatest increase in positioning accuracy has been noted over the last few years for a solution based on several GNSS systems. Multi-system, or multi-GNSS receivers, are currently widely used in smartphones, car navigation systems, sport, recreation, and other mobile applications [18,19,20,21,22,23], including dual-frequency solutions [24,25,26]. Their very rapid development is associated with increasing the positioning accuracy, particularly in urban areas, which results from the rapidly increasing number of satellite systems under construction, i.e., BDS (BeiDou Navigation Satellite System) and Galileo.

Hydrographic surveys are among the navigation applications where GNSS systems are commonly applied. In accordance with the IHO (International Hydrographic Organization) S-44 standard [5], they can be carried out in four orders: special, 1a, 1b, and 2. Each of them was assigned a number of requirements, of which two navigation parameters for positioning systems were primarily defined: maximum allowable positioning accuracy and its confidence level, i.e., the availability of a specific position error value, which is identical for all orders and amounts to 95%.

**Figure 1:**
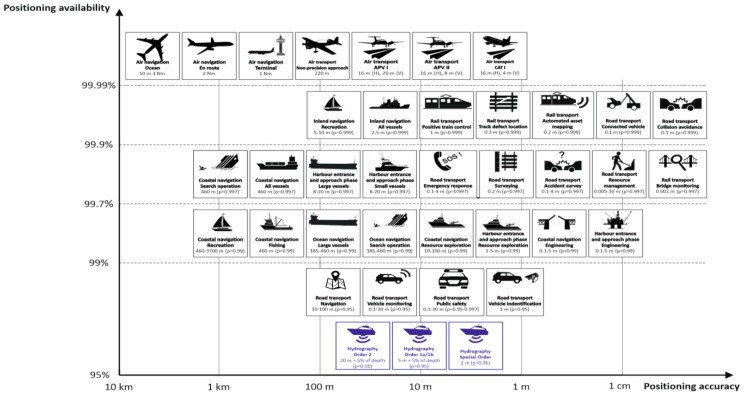
Requirements for positioning system accuracy and availability in hydrography compared to other navigation types. Own study based on: [1,2,3,4,5].

The positioning accuracy and availability requirements imposed on hydrographic operations are among numerous regulations governing the use of positioning systems [27,28,29,30]. Figure 1 shows a synthesis of the requirements imposed on (air, maritime, and land) transport applications as a function of positioning accuracy (the X-axis) and availability (the Y-axis). The diagram was prepared based on an analysis of the data included in global publications defining the requirements for the navigation process, which most frequently were radionavigation plans [1,2,3,4]. The presented graphical proposition was supplemented with the requirements imposed on positioning systems applied in hydrography [5], highlighted in blue.

The analyses conducted in the introduction to this publication indicate that GNSS systems are increasing their positioning accuracy from year to year [31,32]. Given that official characteristics, including those related to the accuracy, for GNSS systems are published by their operators either every few years [10,11,12] or in general (GLONASS (Globalnaja Nawigacionnaja Sputnikowaja Sistiema), BDS, Galileo), the current accuracy value for a particular GNSS solution can only be determined experimentally. For this reason, the determination of how to assess whether a particular system is suitable for a specific application can only be carried out based on actual (stationary and dynamic) measurements, as well as statistical modelling of position error distributions.

In connection with the above, this publication presents a method that enables an assessment of any given navigation positioning system in terms of its compliance (or non-compliance) with the minimum accuracy requirements specified for hydrographic surveys. The proposed model was tested on real measurement data from DGPS and EGNOS systems, as well as multi-GNSS receivers. It should be emphasized that after minor modifications, the presented method can be used for other applications, which are shown in Figure 1.

## 2. Materials and Methods

### 2.1. A Model of Positioning Accuracy and Availability in Hydrographic Surveys According to the IHO Standards

In order to carry out an assessment of the possibility for applying particular positioning methods (DGPS (Differential Global Positioning System), EGNOS (European Geostationary Navigation Overlay Service), and multi-GNSS) in hydrography, a statistical model was proposed that enables the determination of the compliance (or non-compliance) of these three systems with the requirements set out in the IHO S-44 standard [5] and specified by the orders. The model was based on the theory of renewal (repair) process reliability, where the system’s operation and failure statistics are referred to as life and failure times.

Let us consider a positioning system that determines a position with the error $\delta_{n}$ as a function of time, and for which four maximum allowable positioning error values corresponding to the minimum accuracy requirements set out for four IHO orders—special, 1a, 1b and 2—were specified.

**Figure 2:**
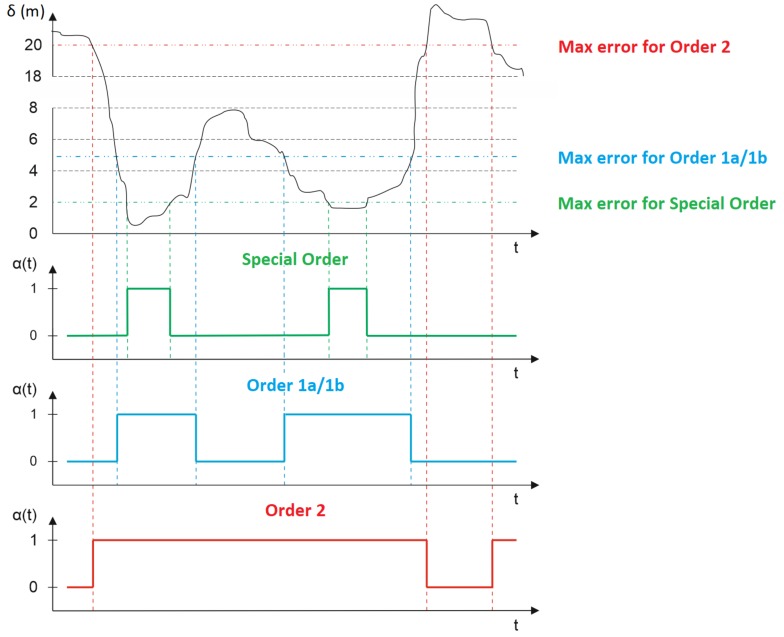
The position error as a function of time (the upper diagram) and three diagrams corresponding to the operational status for the IHO orders: special (green colour), 1a/1b (blue colour), and 2 (red colour).

Figure 2 (the upper diagram) shows a curve that presents the position error value as a function of time conducted by any given positioning system whose assessment in terms of the possibility for use in hydrography should be carried out by assigning an IHO order to it. Let us note that in the presented diagram, since the position error value changes as a function of time, at the beginning it ensures an accuracy of more than 20 m, which prevents it from complying with any of the IHO order requirements. After some time, the position error decreases to just under 2 m, which means that the system can be used for a certain time in all orders, to later reach a value of approximately 8 m, such that it only complies with the requirements set out for order 2. In the diagram, the maximum allowable positioning error values for particular orders: special (green color), 1a/1b (blue color), and 2 (red color) are also plotted.

Under the position error diagram, as a function of time, three diagrams are presented in which the system’s operational status in terms of particular IHO order minimum requirements are shown. From this perspective, a system can have two statuses: fitness (life) designated with a binary value of 1, and unfitness (failure) designated with a binary value of 0. The system’s status is determined using the relationship between the current position error and the maximum allowable positioning error value assigned to a particular IHO order. Three colored diagrams show the system’s operational status in relation to four IHO orders: special, 1a/1b (jointly), and 2.

Thus, the positioning process was transformed to a two-status stationary renewal process. In this way, a transition was made from a random variable, i.e., the position error (a classical approach used in navigation) to a reliability system in which the life and failure times have become random variables. 

In order to determine whether the system has either life or failure status, let us introduce a variable U that corresponds to the maximum allowable positioning error value for four IHO orders, which is to be expressed as follows:

(1) $$U=\left\{\begin{array}{c} 2\;m\;\left(p=0.95\right)\;\mathrm{for}\;\mathrm{special}\;\mathrm{order}\\ 5\;m\;\left(p=0.95\right)\;+\;5\%\;\mathrm{of}\;\mathrm{depth}\;\mathrm{for}\;\mathrm{order}\;1a/1b\\ 20\;m\;\left(p=0.95\right)\;+\;10\%\;\mathrm{of}\;\mathrm{depth}\;\mathrm{for}\;\mathrm{order}\;2 \end{array}\right.$$

**Figure 3:**
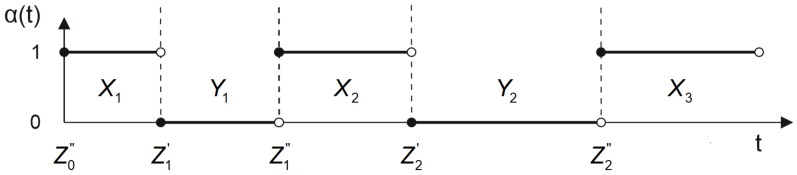
The operational statuses: life (a system’s ability to satisfy the positioning requirements for IHO orders) and failure (an opposite event). Own study based on: [33].

To present the process concerned in a mathematical form, let us assume that the determination of a position’s coordinates is an alternating renewal process, according to the general reliability theory; therefore, it can be assigned two statuses as a function of time: life, i.e., a status in which the position error is smaller than the arbitrarily established value corresponding to the requirements of particular IHO orders, which is to be expressed as $\delta_{n}\;\leq \;U$ for the subsequent determinations of n = 1, 2, …, and the failure time in which an opposite relationship $\delta_{n}\;>\;U$ occurs. Let us also assume that the values $X_{1},\;X_{2},\;\ldots$ correspond to the duration of life times (a position error below the allowable value according to the IHO orders), and $Y_{1},\;Y_{2},\;\ldots$ correspond to their failure times (a position error above the allowable value according to the IHO orders). In this way, a consequence of the change in the position error is a change in the operational status of a system represented by variable $\alpha \left(t\right)$(Figure 3). Let us also introduce additional designations so that the moments of time $Z_{n}^{\prime }\;=\;X_{1}\;+\;Y_{1}+\;X_{2}\;+\;Y_{2}+\;\ldots \;+\;Y_{n-1}{\;+\;X}_{n}$ become moments of failure, while moments $Z_{n}^{"}\;=\;Z_{n}^{\prime }{\;+\;Y}_{n}$ are moments of renewal. Let us additionally assume that the random variables $X_{i},\;Y_{i}$ for i = 1, 2, … are independent, and that the life and failure times have identical distributions.

For the model, it is necessary to introduce several additional designations and assumptions resulting from its mathematical properties [34]. Let us assume that the distribution functions of life times $F\left(x\right)$ and failure times $G\left(y\right)$ are right-continuous. Then:

(2) $$P\left(X_{i}\leq x\right)=F\left(x\right)\;$$

(3) $$P\left(Y_{i}\leq y\right)=G\left(y\right)\;\;\mathrm{for}\;i=1,2,\ldots$$

Let us also introduce designations of the expected value and variance, which can be expressed as:

(4) $$E\left(X_{i}\right)=E\left(x\right)\;$$

(5) $$E\left(Y_{i}\right)=E\left(y\right)\;$$

(6) $$V\left(X_{i}\right)=\sigma_{1}^{2}\;$$

(7) $$V\left(Y_{i}\right)=\sigma_{2}^{2}\;\;\mathrm{for}\;i=1,2,\ldots$$

where:

$E\left(X_{i}\right)$—life time expected value,

$E\left(Y_{i}\right)$—failure time expected value,

$V\left(X_{i}\right)$—life time variance, and

$V\left(Y_{i}\right)$—failure time variance.

Moreover, let us also assume that:

(8) $$\sigma_{1}^{2}+\sigma_{2}^{2}>0$$

Based on the adopted assumptions, let us establish the reliability process in which its status (of either life or failure) is determined by the relationship between a single measurement error $\delta_{n}$ and the parameter U assigned to a particular IHO order. Let $\alpha \left(t\right)$ be a binary interpretation of the reliability status of this process in the following form:

(9) $$U=\left\{\begin{array}{l} 1\;\mathrm{for}\;Z_{n}^{"}\leq t<Z_{n+1}^{\prime }\\ 0\;\mathrm{for}\;Z_{n+1}^{\prime }\leq t<Z_{n+1}^{"} \end{array}\right.\;\mathrm{for}\;n=0,1,\ldots$$

The status $\alpha \left(t\right)\;=\;1$ denotes that at the moment t, the single measurement error was smaller than or equal to the value of the allowable position error U determined according to Equation (1). Otherwise, for $\delta_{n}\;>\;U$, let us assume that the system has a failure status.

To be able to determine a positioning system’s availability corresponding to the defined position error value of the analyzed system, let us define this value as the probability that at any given moment of time t, the position’s error $\delta_{n}$ will be smaller than or equal to the arbitrarily adopted value U. Let us designate it with variable $A\left(t\right)$ while assigning to it a mathematical formula in the following form:

(10) $$A\left(t\right)=P\left[\delta \left(t\right)\leq U\right]$$

(11) $$A\left(t\right)=1-F\left(t\right)+\int_{0}^{t}\left[1-F\left(t-x\right)\right]dH_{\Phi }\left(x\right)$$

where:

(12) $$H_{\Phi }\left(x\right)=\sum_{n=1}^{\infty }\Phi_{n}\left(x\right)$$

is a function of the renewal stream made up of the renewal moments of the navigation system complying with a specific IHO order, while $\Phi_{n}\left(t\right)$ is a distribution function of the random variable $Z_{n}^{"}$.

**Figure 4:**
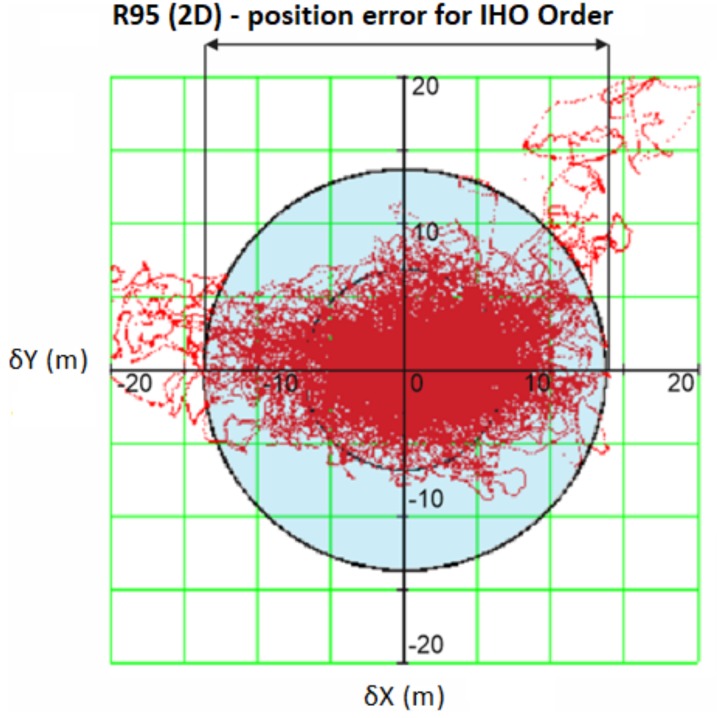
An example of geometric interpretation of the availability required for IHO order 2.

The concept of the method can also be presented in the graphical form (Figure 4) where the figure shows the distribution of subsequent positions of the analyzed system in relation to the actual values (the center of the diagram). In the diagram, a circle with a radius of 20 m is marked. This radius corresponds to the maximum allowable position error value for IHO order 2, which means that the positions within the circle correspond to the condition $\delta_{n}\;\leq \;U$, i.e., the system has a life status, while the positions located outside the circle correspond to the system’s failure statuses.

In navigation applications, it is most frequently assumed that the distributions of life and failure times are exponential, hence their probability density functions can be expressed using commonly known formulas [34]:

(13) $$f\left(t\right)=\left\{\begin{array}{c} \lambda \cdot e^{-\lambda \cdot t}\;\mathrm{for}\;t>0\\ 0\;\mathrm{for}\;t\leq 0 \end{array}\right.$$

(14) $$g\left(t\right)=\left\{\begin{array}{c} \mu \cdot e^{-\mu \cdot t}\;\mathrm{for}\;t>0\\ 0\;\mathrm{for}\;t\leq 0 \end{array}\right.$$

with the following respective distribution functions:

(15) $$F\left(t\right)=\left\{\begin{array}{c} 1-e^{-\lambda \cdot t}\;\mathrm{for}\;t>0\\ 0\;\mathrm{for}\;t\leq 0 \end{array}\right.$$

(16) $$G\left(t\right)=\left\{\begin{array}{c} 1-e^{-\mu \cdot t}\;\mathrm{for}\;t>0\\ 0\;\mathrm{for}\;t\leq 0 \end{array}\right.$$

where:

$f\left(t\right)$—life time probability density function,

$g\left(t\right)$—failure time probability density function,

λ—failure rate,

μ—renewal rate.

Having adopted the above assumptions, the final form of the availability function can be expressed as:

(17) $$A_{\exp}\left(t\right)=\frac{\mu }{\lambda +\mu }+\frac{\lambda }{\lambda +\mu }\cdot e^{-\left(\lambda +\mu \right)\cdot t}$$

where $A_{\exp}\left(t\right)$ is an availability function determined for a specific position error value according to the IHO orders, with the assumed exponential distribution of the positioning system’s life and failure times.

In practical applications, it is advisable to provide the availability limit value $\left(A_{\exp}\right)$ [9], referred to as the availability factor [34]:

(18) $$\underset{t\to \infty }{\lim}\left[A_{\exp}\left(t\right)\right]=A_{\exp}=\frac{\frac{1}{\lambda }}{\frac{1}{\lambda }+\frac{1}{\mu }}=\frac{\mu }{\mu +\lambda }$$

### 2.2. A Measurement Assessment of the Compliance of DGPS, EGNOS, and Multi-GNSS with the Positioning Requirements for the IHO orders

**Figure 5:**
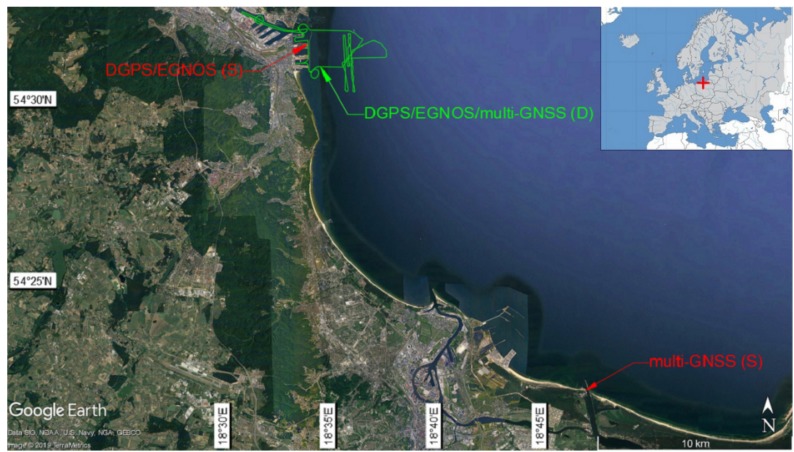
The place of dynamic (D) and stationary (S) measurements of the DGPS and EGNOS systems, as well as multi-GNSS receivers.

The assessment of particular positioning systems (DGPS, EGNOS, and multi-GNSS) in terms of compliance with the requirements set out for the IHO orders should be carried out based on measurement sessions that are as long as possible. In addition, it should be noted that the results of an accuracy assessment for a certain system in both stationary and dynamic sessions may be significantly statistically different, which is proven by the research carried out on the Gdańsk Bay in the years 2014–2017 (Figure 5) [35,36,37,38]. For this reason, analyses and assessments of the model should be carried out in relation to stationary and dynamic measurements in parallel.

To carry out the analyses and calculations in accordance with the proposed model, the data were gathered from:

- Stationary measurements of the DGPS and EGNOS systems carried out on the radio beacon in the port of Gdynia in April and May 2014. As part of the study, two receivers were used: Leica MX-Marine (DGPS) and Topcon Legacy-E (EGNOS), which simultaneously recorded their position coordinates with a frequency of 1 Hz. During the several-day measurement campaign, the accuracy statistics of these systems were determined based on nearly 1 million positions (Table 1) [35].
- Dynamic measurements of the DGPS and EGNOS systems carried out during vessel manoeuvring on the Gdańsk Bay on 20 June 2017. As part of the study, two receivers were used: Simrad MXB5 (DGPS) and Trimble GA530 (EGNOS), which simultaneously recorded their position coordinates with a frequency of 1 Hz. The obtained values were compared with the precise GNSS receivers (Trimble R10), using corrections from an active geodetic network with an accuracy of 2–3 cm (*p* = 0.95). During a 4-hour measurement session, the accuracy statistics of these systems were determined based on approximately 11,500 positions (Table 1) [37].
- Stationary measurements of the multi-GNSS systems using the example of Samsung Galaxy smartphones, carried out on the rooftop of the National Sailing Centre in Gdańsk on 17 July 2017. As part of the study, the following smartphones were used: S2, S3 Mini, S4, S5, S6, S7, and Y, whose coordinates (antennas) were determined using geodetic methods, and with their use, a round-the-clock measurement campaign was carried out. It consisted of parallel registration of position coordinates by all telephones. During a 24-hour measurement session, between 71,438 and 86,371 positions were registered depending on the smartphone model (Table 2) [38].
- Dynamic measurements of the multi-GNSS systems using the example of Samsung Galaxy smartphones, carried out during vessel manoeuvring on the Gdańsk Bay on 20 June 2017. For the comparative analysis, five Samsung Galaxy S series smartphones were used, namely: 3 Mini, 4, 5, 6, 7, and one Galaxy Y. As part of the parallel tracking studies, the telephone positions were compared to those of precise GNSS receivers (Trimble R10) using corrections from an active geodetic network with an accuracy of 2–3 cm (*p* = 0.95). As a result of the 4-hour measurement, the accuracy statistics for each of the phone models were defined based on approximately 10,000 positions (Table 3) [36].

The data originating from multi-GNSS receivers were handled in a similar manner. For the analyses, measurement data from both the stationary and dynamic campaigns described in detail in References [36,38] were used. These are measurement data originating from five Samsung Galaxy S series smartphones, designated successively with the following numbers: 3 Mini, 4, 5, 6, 7, and Galaxy Y. The final results of classical accuracy analyses are presented in Table 2 (stationary measurements) and Table 3 (dynamic measurements).

The results of tests on the DGPS and EGNOS systems, as well as the multi-GNSS receivers presented in the tables, refer to a confidence level of 0.95 where position errors corresponding to this value were determined. They were obtained using two commonly applied methods, i.e., through the determination of a standard deviation with an assumed normal distribution of errors and through grading the errors from the lowest to the highest value in order to determine the R95 value.

## 3. Results and Discussion

**Figure 6:**
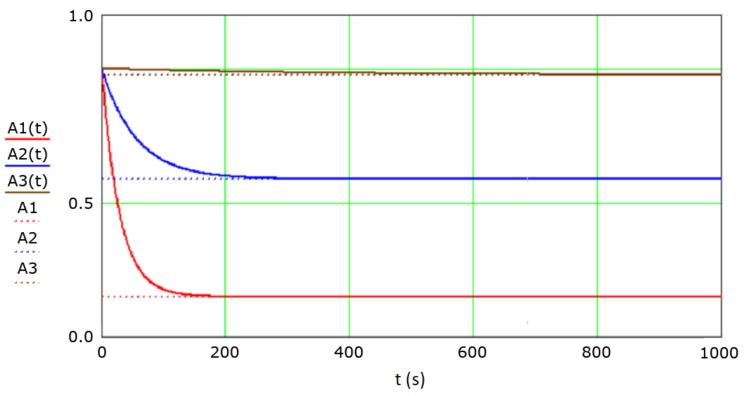
Availability functions: *A1*(*t*), *A2*(*t*), and *A3*(*t*) and their limit values: *A1*, *A2*, and *A3*. These correspond to four IHO orders: special (red colour), 1a/1b (blue colour), and 2 (brown colour).

The mathematical model from Chapter 2 was implemented in the Mathcad 15 software, which conducted statistical calculations and analyses using the data from the measurement campaigns mentioned in the previous subchapter. For each of the IHO orders (which are characterized by different allowable position errors), the availability function value was calculated. Figure 6 shows an example of three availability functions: $A1\left(t\right),\;A2\left(t\right),\;A3\left(t\right)$, determined based on Equation (17), as well as their limit values: A1, A2, A3, calculated based on Equation (18) for the maximum allowable position errors corresponding to the IHO orders: special (2 m), 1a/1b (5 m), and 2 (20 m). Diagrams of the availability functions for the orders 1a and 1b were combined because of the identical requirements.

Analogous calculations were conducted for the measurement results of the DGPS and EGNOS systems, as well as multi-GNSS receivers (installed in Samsung Galaxy smartphones). The summary results are listed in Table 4.

The analysis of the DGPS system testing results for stationary and dynamic measurements clearly demonstrates that the system complied with all the requirements set out for all IHO orders. Hence, it can be applied while carrying out hydrographic surveys, irrespective of the order. The results presented are consistent with results of studies by other authors [39].

Having compared the DGPS and EGNOS solutions, it should be noted that the DGPS system ensured a slightly greater availability than the EGNOS system did in terms of hydrographic survey needs. What needs an additional comment is the result for the availability of the EGNOS system (Table 4), which in the course of stationary measurements, amounted to 89.11% for the IHO special order without reaching the minimum value (95%). In the author’s opinion, since the presented multi-annual research results prove that this system permanently improves the positioning characteristics, its use in hydrography will soon be possible for all IHO orders. A significant increase in the EGNOS system’s accuracy over recent years has also been noted by other authors [40,41] and official regulations [42]. Despite this fact, the EGNOS system is still not used in hydrography [43], as one would expect.

**Figure 7:**
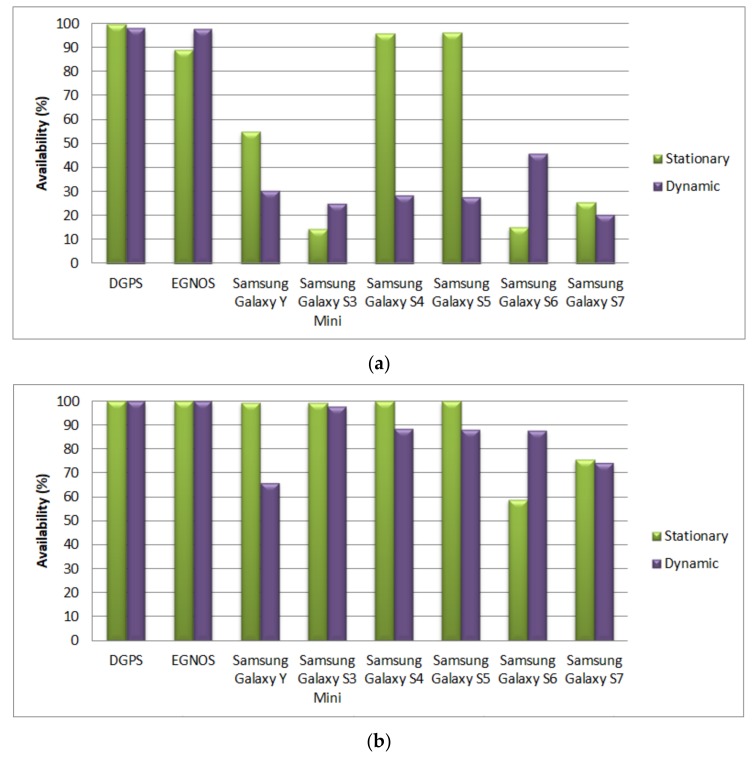
The availability of the DGPS and EGNOS systems, as well as multi-GNSS receivers, for the IHO special order (**a**) and for the order 1a/1b (**b**).

An unambiguous assessment of the possibility for using multi-GNSS receivers in hydrography requires an alternative form of result presentation due to significant differences in the results of stationary and dynamic tests. Figure 7 presents diagrams of the availability of particular positioning solutions for both stationary and dynamic measurements, dividing them into two orders: special and 1a/1b. The final order, order 2, required no separate analysis as the interpretation of results from Table 4 is clear.

While assessing multi-GNSS receivers in the context of the possibility for ensuring the required accuracy for the special order, one should note the significant difference in the results of both stationary and dynamic measurements, particularly for models S4 and S5. Since the performance of hydrographic surveys is related to dynamic measurements, it can be concluded that none of the receivers installed in smartphones complied with the requirements of this order. On the other hand, for the order 1a/1b, models S4 and S5 (with dual-system GPS/GLONASS modules) deviated only slightly from the minimum availability requirements (95%). In the analyzed dynamic tests, the availability factor for models S4 and S5 reached 88.28% and 88.68%, respectively.

It should be very clearly stressed that in order to present the proposed method, this article used measurement data from various tests on DGPS and EGNOS systems and multi-GNSS receivers. The method presents a new approach to the assessment of the systems’ possibilities in terms of the IHO requirements, since for the assessment of the system’s accuracy and availability, mathematical models based on life and failure times were used instead of the position errors typically adopted in navigation. The fact that the analyses were carried out on data originating from different years is of no significance here, as the purpose of this publication is to present the original author’s method. The above-mentioned results of the measurement campaigns enabled the verification of the model based on actual data.

## 4. Conclusions

The article proposes a method that enables an assessment of any given navigation positioning system in terms of its compliance (or non-compliance) with the minimum accuracy requirements specified for hydrographic surveys. The unique feature of the model is the possibility of carrying out an assessment based on actual measurements and analyses of a positioning system’s operating and failure times. In the example calculations, it was assumed that the distributions of operating and failure times are exponential (in accordance with Equations (13)–(18)). However, where sufficient series of measurements of the analyzed system are available, it is possible to determine through measurements the actual statistical distribution parameters and their distribution functions while enabling the application of Equation (11), which describes the system’s positioning process, in statistical terms, in more detail. The presented model was applied to three different selected GNSS solutions, i.e., DGPS and EGNOS systems, as well as multi-GNSS receivers. Based on analyses using very extensive and real measurement data, it has been shown that the proposed model can be successfully used in practice.

It should be noted that the proposed method is not limited only to autonomous and augmentation GNSS systems. It can be successfully applied to other positioning systems that require the indication of the range of their use in various navigation types [44,45].

The proposed method can also be applied outside of hydrography. With minor modifications, it can be successfully applied to all positioning applications listed in Figure 1.

## Figures and Tables

**Table 1:** Statistics for the DGPS and EGNOS systems’ predicted position errors during the course of the stationary (S) and dynamic (D) measurement campaigns [35].

Statistics of Position Error	DGPS 2014 (S)	EGNOS 2014 (S)	DGPS 2017 (D)	EGNOS 2017 (D)
Number of measurements	951,698	927,553	11,751	11,698
2DRMS (2D)	0.96 m	3.27 m	1.48 m	2.39 m
R95 (2D)	0.83 m	2.31 m	1.42 m	1.79 m

**Table 2:** Statistics for the Samsung Galaxy phones’ predicted position errors over the course of a 24-hour stationary measurement campaign [38].

Statistics of Position Error	Samsung Galaxy Series					
	Y	S3 Mini	S4	S5	S6	S7
Number of measurements	73,699	71,438	86,290	86,346	86,371	86,355
2DRMS (2D)	5.61 m	6.79 m	2.04 m	2.06 m	13.69 m	8.93 m
R95 (2D)	4.93 m	3.76 m	1.65 m	1.76 m	12.64 m	8.39 m

**Table 3:** Statistics for the Samsung Galaxy phones’ predicted position errors over the course of a dynamic measurement campaign [36].

Statistics of Position Error	Samsung Galaxy Series					
	Y	S3 Mini	S4	S5	S6	S7
Number of measurements	6,041	3,410	10,950	10,939	10,906	10,926
2DRMS (2D)	9.47 m	5.23 m	6.59 m	6.72 m	8.32 m	10.54 m
R95 (2D)	6.84 m	3.67 m	5.80 m	5.77 m	9.38 m	9.62 m

**Table 4:** A summary of the availability factor values, determined for the DGPS and EGNOS systems, as well as multi-GNSS receivers in the context of minimum positioning requirements determined for four IHO orders. The measurements relate to stationary and dynamic tests.

Year.	System	Special Order		Order 1a/1b		Order 2	
		Availability		Availability		Availability	
		Stationary	Dynamic	Stationary	Dynamic	Stationary	Dynamic
**2014 (S)/2017 (D)**	DGPS	**99.76**	**98.09**	**100**	**100**	**100**	**100**
2014 (S)/2017 (D)	EGNOS	**89.11**	**97.52**	**100**	**100**	**100**	**100**
2017	Samsung Galaxy Y	**54.88**	**30.73**	**99.39**	**66.02**	**100**	**100**
2017	Samsung Galaxy S3 Mini	**14.39**	**25.52**	**99.16**	**97.85**	**100**	**100**
2017	Samsung Galaxy S4	**95.7**	**28.67**	**99.98**	**88.68**	**100**	**100**
2017	Samsung Galaxy S5	**96.36**	**27.92**	**100**	**88.28**	**100**	**100**
2017	Samsung Galaxy S6	**14.99**	**45.92**	**58.82**	**87.7**	**97.54**	**99.45**
2017	Samsung Galaxy S7	**25.35**	**20.84**	**75.38**	**74.21**	**99.76**	**98.67**
